# Supplementary material for: Consequences of Structural Urbanism: Urban–Rural Differences in Cancer Patients’ Use and Perceived Importance of Supportive Care Services from a 2017–2018 Midwestern Survey
Source: Int J Environ Res Public Health. 2022 Mar 14;19(6):3405. doi: 10.3390/ijerph19063405 (PMC8955585; doi:10.3390/ijerph19063405)
Supplement: Supplementary file 1 [file ijerph-19-03405-s001.zip › ijerph-1590004-supplementary.pdf]

Supplementary Materials:

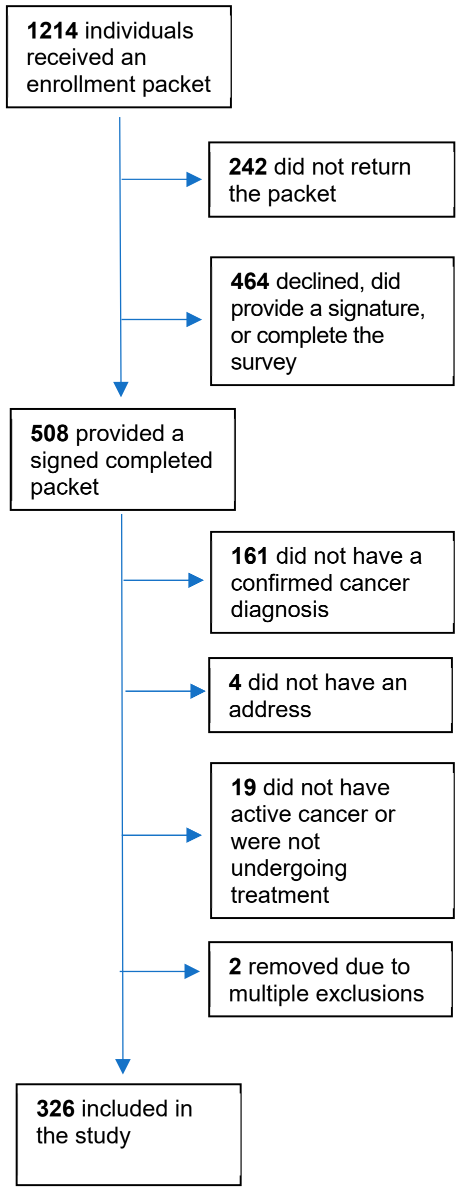

Figure S1: Flow diagram of participants of the sectional needs assessment about patients' support needs, use of services, and perceptions at a cancer center.

Table S1: Descriptive statistics regarding specific severe support needs, use of specific support services, and perceived importance of specific support services

	Urban (n = 238)		Rural (n = 88)		Overall (n = 326)	
	N	%	N	%	N	%
**Specific Severe Support Needs**						
Work	84	35%	39	34%	123	38%
Weak	73	31%	30	34%	103	32%
Intimacy	72	30%	30	34%	102	31%
Pain	52	22%	20	23%	72	22%
Sleep	41	17%	15	17%	56	17%
Mouth/taste	36	15%	15	17%	51	16%
Appetite	35	15%	10	11%	45	14%
Ability for activities	32	13%	17	19%	49	15%
Future	28	12%	11	13%	39	12%
Short Breath	28	12%	8	9%	36	11%
Concentration	26	11%	9	10%	35	11%
Mood	25	11%	14	16%	39	12%
Finances	25	11%	12	14%	37	11%
Worries about others	25	11%	9	10%	34	10%
Constipation	20	8%	4	5%	24	7%
Tense/worried/fearful	19	8%	13	15%	32	10%
Self-Care	19	8%	8	9%	27	8%
Bladder	16	7%	8	9%	24	7%
Communication	16	7%	8	9%	24	7%
Irritable	15	6%	11	13%	26	8%
Worried appearance	15	6%	10	11%	25	8%

Swallowing	15	6%	8	9%	23	7%
Treatment side effects	14	6%	9	10%	23	7%
Lack of information	12	5%	6	7%	18	6%
Relationships	12	5%	4	5%	16	5%
Lack of support	11	5%	6	7%	17	5%
Feeling sick	10	4%	7	8%	17	5%
Diarrhea	10	4%	7	8%	17	5%
Spiritual	5	2%	3	3%	8	2%
**Supportive Services Use**						
None	173	73%	75	85%	248	76%
Any	65	27%	13	15%	78	24%
**Specific Services Use**						
Patient navigation	40	17%	10	11%	50	15%
Counseling	24	10%	3	3%	27	8%
Genetic services	20	8%	5	6%	25	8%
Support groups	19	8%	2	2%	21	6%
Smoking cessation program	17	7%	1	1%	18	6%
Wellness program	13	6%	1	1%	14	4%
Fertility services	12	5%	1	1%	13	4%
**Importance of Services**						
None	85	36%	43	49%	128	39%
Any	153	64%	45	51%	198	61%
**Importance of Specific Services**						
Pain management	101	42%	21	24%	122	37%
Nutritional education	86	36%	24	27%	110	34%

Advance care planning	79	33%	14	16%	93	29%
Rehabilitation	76	32%	18	21%	94	29%
Survivorship planning	74	31%	21	24%	95	29%
Integrative therapy	68	29%	14	16%	82	25%
Financial counseling	63	27%	16	18%	79	24%
Self-serve resources	63	27%	12	14%	75	23%
Educational programs	63	27%	12	14%	75	23%
Spiritual counseling	50	21%	11	13%	61	19%
Psychiatric medication	42	18%	12	14%	54	17%
Sexual health services	38	16%	6	7%	44	13%
Travel/lodging assistance	38	16%	20	23%	58	18%
Child life specialist services	30	13%	5	6%	35	11%
